# Supplementary material for: Genomic Characterization and Expression Analysis of the SnRK Family Genes in Dendrobium officinale Kimura et Migo (Orchidaceae)
Source: Plants (Basel). 2021 Mar 3;10(3):479. doi: 10.3390/plants10030479 (PMC8000535; doi:10.3390/plants10030479)
Supplement: Supplementary file 1 [file plants-10-00479-s001.zip › Supplementary files/Figure S1.pdf]

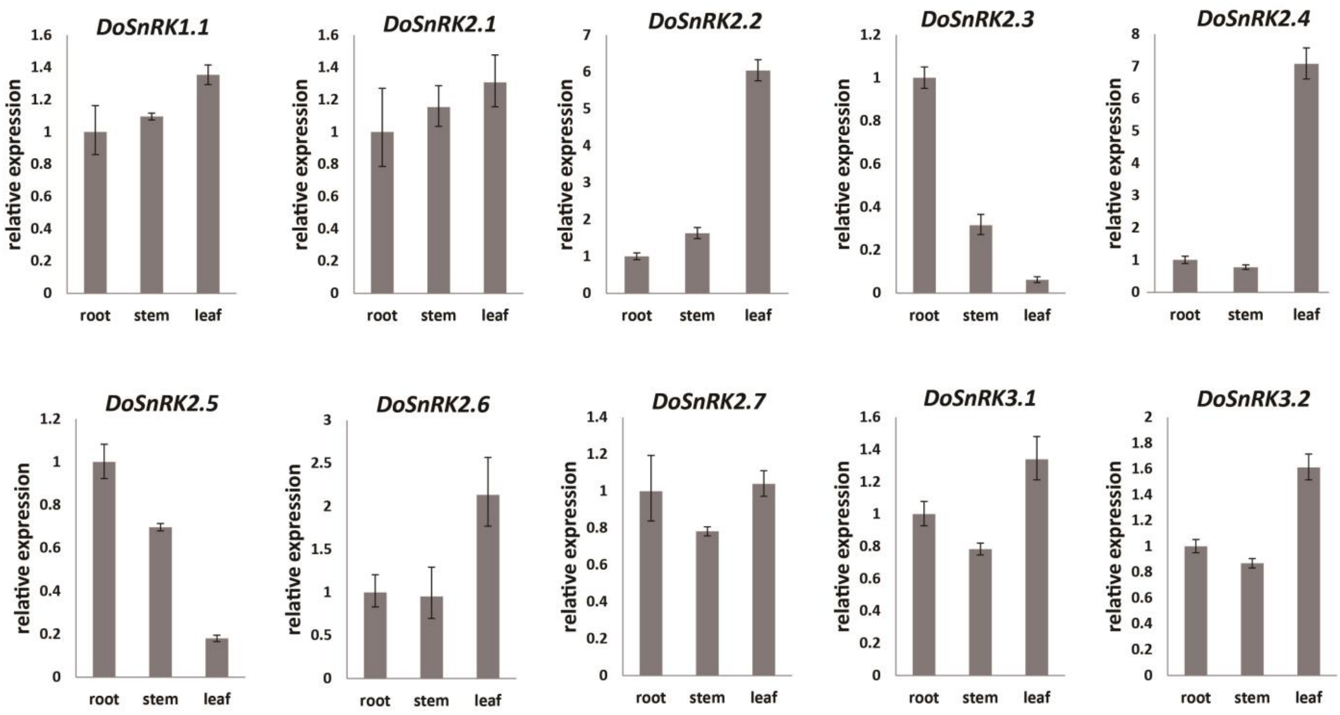

Figure S1. Expression profiles of 10 *DoSnRK* genes in leaf, stem, and root tissues by qRT-PCR assay. The relative expression levels were obtained from the average of three repeats. The bars stand for the standard deviation.
